# Photodynamic Therapy of Up-Conversion Nanomaterial Doped with Gold Nanoparticles

**DOI:** 10.3390/ijms23084279

**Published:** 2022-04-13

**Authors:** Wei Zhang, Yang Zang, Yanli Lu, Jinghui Han, Qingyun Xiong, Jinping Xiong

**Affiliations:** 1 Beijing Key Laboratory of Electrochemical Process and Technology of Materials, Beijing University of Chemical Technology, Beijing 100029, China; 2019310030@mail.buct.edu.cn (Y.Z.); 2019310036@mail.buct.edu.cn (Y.L.); 2020200494@mail.buct.edu.cn (Q.X.); 2 State Key Laboratory of Organic-Inorganic Composites, Beijing University of Chemical Technology, Beijing 100029, China; 2021400008@buct.edu.cn

**Keywords:** up-conversion, nanomaterials, photodynamic, photothermal

## Abstract

Two key concerns exist in contemporary cancer chemotherapy: limited therapeutic efficiency and substantial side effects in patients. In recent years, researchers have been investigating the revolutionary cancer treatment techniques of photodynamic therapy (PDT) and photothermal therapy (PTT) proposed by many scholars. A photothermal treatment of cancer was synthesized using the hydrothermal method which has high photothermal conversion efficiency and can generate reactive oxygen species (ROS) in cells. Photothermal treatment of tumors has a good short-term effect and photodynamic therapy lasts longer. However, both PTT and PDT have their inevitable shortcomings and it is difficult to completely eradicate a tumor using a single mode of treatment. PTT and PDT synergistic treatment not only inherits the advantages of low toxicity and side effects of phototherapy but also enables the two treatment methods to complement each other. It is an effective strategy to improve curative effects and reduce toxic and side effects. Furthermore, gold doped UCNPs have an exceptionally high target recognition for tumor cells. The gold doped UCNPs, in particular, are non-toxic to normal tissues, endowing the as-prepared medications with outstanding therapeutic efficacy and exceptionally low side effects. These findings may encourage the creation of fresh, effective imaging-guided approaches to meet the goal of photothermal cancer therapy.

## 1. Introduction

Interest in developing theranostic nanoplatforms with simultaneous diagnostic and therapeutic capacity has gradually increased in the nanomedicine field because they provide significant prospects in the treatment of major illnesses, including cancer [1]. Imaging probes, as one of the most important components of the theranostic nanoplate-form, should be able to perform many levels of imaging at the same time, from the cell to the whole body, to offer comprehensive tumor characteristics for clinical diagnostics. However, no matter which imaging technique (optical imaging, CT, or MRI) is used, it has intrinsic flaws due to restricted resolution, sensitivity, or imaging depths; a single treatment method can not match the high treatment criteria since each treatment method (chemotherapy, radiotherapy, photothermal therapy, and photodynamic therapy) has intrinsic flaws due to their different treatment principles.

To mitigate this problem, two treatments were combined into a single nanosystem with some considerable restrictions, such as sophisticated synthetic processes and heterogeneous nanostructures. As a result of their enhanced optical, low background, and deep tissue penetration properties, lanthanide-doped upconverting nanoparticles (UCNPs) might be perfect for building multifunctional nanoplatforms by doping with various rare-earth ions without modifying other functions.

Many researchers have recently advocated that UCNPs be employed in biological imaging since they provide considerable benefits in the treatment of major illnesses such as cancer. However, the typical challenge is that they have insufficient light intensity and are poisonous to biological cells, therefore their structure and surface must be modified [2]. Many scholars have proposed doping Mo^3+^, Cu^2+^ [3,4], and other metal ions in the NaYF_4_:Yb^3+^ / Er^3+^ unit cell to increase the luminous intensity [5,6], but the effect is not significant. Others have offered sliver doping [7], which has a large impact as well; however, sliver is poisonous to cells, may cause cell death without targeting, and cannot be employed in biology. Many scholars have proposed constructing core-shell structures such as NaYF_4_:Yb^3+^/Er^3+^@NaGdF_4_:Yb^3+^ and NaYF_4_:Yb^3+^/Er^3+^@NaNdF_4_:Yb^3+^/Tm^3+^@NaGdF_4_:Yb^3+^ [8,9,10]. Alternatively, using the reverse microemulsion method to construct a layer of silica or porous silica, such as NaYF_4_:Yb^3+^ /Er^3+^@SiO_2_, NaYF_4_:Yb^3+^/Er^3+^@NaGdF_4_:Yb^3+^@m-SiO_2_ [11,12,13,14]. Gold nanoparticles are currently the mainstream biomaterials in tumor diagnosis and treatment applications [15,16]. They are widely used in CT imaging and photoacoustic imaging due to their excellent imaging capabilities and photothermal effects [17]. However, in our research, their photothermal stability does not seem to be very good [18]. Unfortunately, once these materials meet the biological requirements, they will inevitably reduce all aspects of performance, which greatly reduces their efficiency [19,20]. Considering the high desire to develop the efficient therapeutic ability of UCNPs with the integration of tumor diagnosis and treatment, simplifying the treatment of cancer, the UCNPs doped with gold nanoparticles are an ideal choice because they not only generate ROS under the near-infrared light irradiation at the 808 nm wavelength but also generate heat energy under the near-infrared light irradiation at the 980 nm wavelength [21]. These two wavelengths can penetrate biological tissues and these two effects have great inhibitory effects on tumor cells. More importantly, due to the excellent photothermal properties of this material, it can be used for PAI. Compared with common CT and MRI, PAI has a clearer effect and can be used for nondestructive tissue imaging. However, to the best of our knowledge, there appears to be a failure in the literature to create theranostic nanoplatforms integrating multi-modality bioimaging with light trigger chemotherapy.

## 2. Experiment

### 2.1. Materials

The Au nanoparticles and (Au doped with Up-conversion nanoparticles were modified by DSPE-PEG_2K_ (Distearoylphosphatidylethanolamine-polyethylene glycol_2K_)) Au-UCNPs-DSPE-PEG_2K_ were synthesized in Beijing Key Laboratory of Electrochemical Process and Technology of Materials, Beijing University of Chemical Technology [22]. Hela cells and BALB/c female white mice with SPF grade came from Beijing Laboratory of Biomedical Materials, Beijing University of Chemical Technology. The cell counting kit 8 (CCK-8) assay kit was acquired from BOVOGEN (Beijing, China). Dulbecco’s modified eagle’s medium (DMEM), Singlet Oxygen Sensor Green (SOSG), and Distearyl phosphatidylethanolamine polyethylene glycol (DSPE-PEG_2K_) were acquired from Aladdin (Shanghai, China). All chemicals were utilized in as-received condition, without further refinement.

### 2.2. Characterization of Materials

A spectrum analyzer (ANDO AQ6317, Yokohama, Japan) was used to get the up-conversion luminescence spectra. The specimen was positioned in a 1.0 cm path length support and excited by utilizing a 980 nm CW semiconductor diode laser (Pmax 800 mW, 1000 mA). The up-conversion luminescence spectrum was acquired through the spectrophotometer having a multimode fiber with a core diameter of 0.6 mm. The top of the fiber was ~2 mm away from the specimen. A thermal imager (FOCUS 280DS, Beijing, China) was used to characterize the photoacoustic properties of photographic materials. A HORIBA (Beijing, China) laser and power density meter were used to characterize photothermal properties.

### 2.3. CCK-8 Assay for Cytotoxicity

HeLa cells were cultured in the logarithmic growth phase, and the culture medium was sucked out from the flask. The cells were then washed with PBS and digested with the help of 0.25% trypsin. Then the trypsin was removed, and the cells were blown with DMEM media containing 10% fetal bovine serum before being shifted to the sample tank and blown well. Following that, 100 µL cells were introduced onto a 96-well plate (1 × 10^4^ cells/well) and cultured for 24 h at 37 °C in a constant temperature incubator (5% CO_2_). The cells were cultured in an incubator at 37 °C with 5% CO_2_ for 1.5 h at concentrations of 200, 300, 400, 500, and 600 µg/mL of Au-UCNPs-DSPE-PEG_2K_, respectively. The culture media was blotted out, PBS was washed twice, and the culture medium in the 96-well plates was replaced with 100 µL of fresh DMEM containing 10% fetal bovine serum, followed by 10 µL of CCK-8 solution in each well. After 2 h of incubation, the absorbance of each well at 450 nm was measured with a microplate reader. The formula for calculating cell survival rate is as follows:

Cell survival rate (%) = (A specimen)/(A control) × 100%

### 2.4. SOSG Assay for ROS

100 µg SOSG was dissolved in 6600 µL of an oxygen-free methanol solution and prepared as a mother liquor with a concentration of 15 µg/mL, then stored away from light for later use. A sample solution (200 µg /mL) was prepared, 100 µL withdrawn and mixed in a 96-well plate with 50 µL of the SOSG mother liquor and then irradiated with near-infrared light with a wavelength of 808nm (irradiance 0.5 W/cm^2^) for 0, 10, 20, 30, 40 min with each concentration set to three repeated values. Finally, the spectrometer measured the fluorescence intensity of SOSG at 525nm and detected other concentrations (300, 400, 500, 600 µg/mL) in the same way.

## 3. Results and Discussion

**Figure 1:**
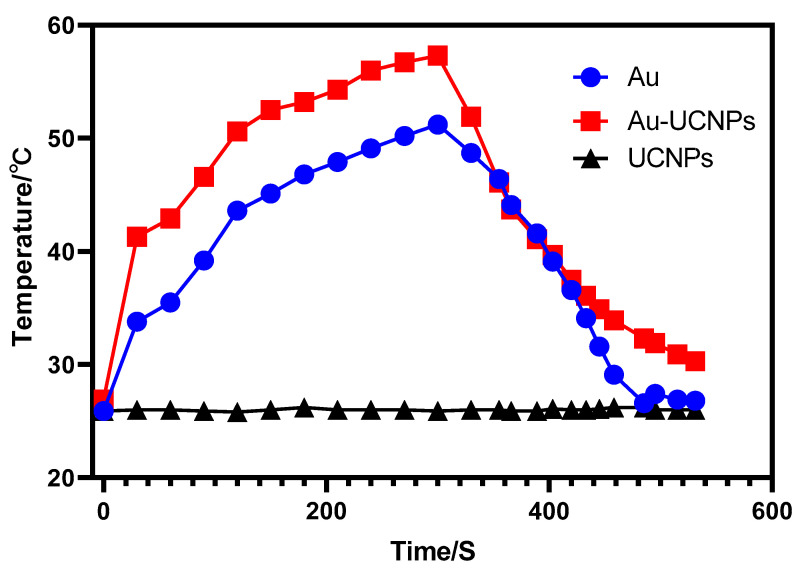
Temperature rise and drop curves of gold nanoparticles (blue) irradiated by near-infrared light at a 540 nm wavelength; UCNPs (black) and Au-UCNPs (red) irradiated by near-infrared light at a 980 nm wavelength.

**Figure 2:**
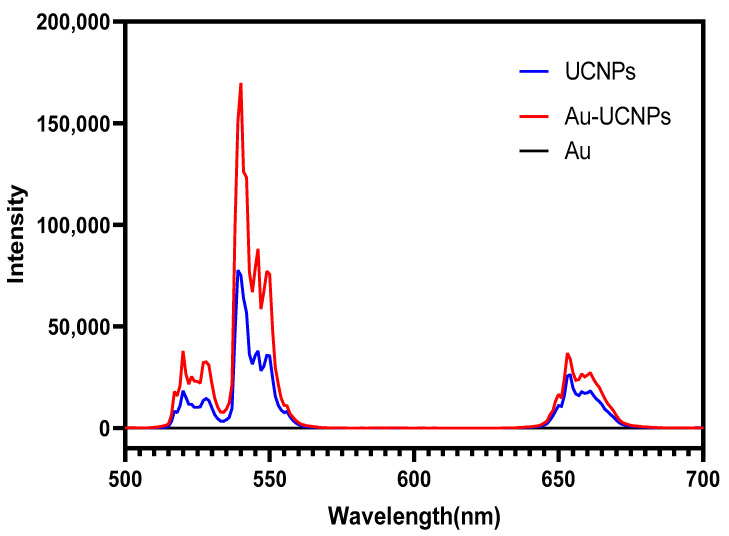
Luminescent intensity of UCNPs (blue), Au (black), and Au-UCNPs (red).

**Figure 3:**
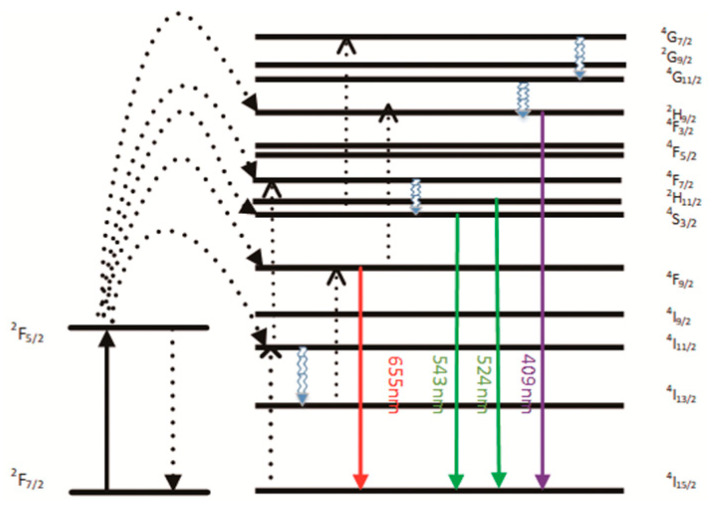
A simplified energy level diagram of the Er3+/Yb3+ system doped with Au, and up-conversion pathways. The arrows represent the direction of electron migration, and the dashed and solid lines of the arrows represent the energy levels of the electron transitions. Arrows of different colors represent different wavelengths.

The gold nanoparticles exhibit a temperature increase phenomenon when exposed to a laser with a wavelength of 540 nm and a power of 500 mW (Figure 1). The irradiation ends when the temperature reaches 52 °C and the gold nanoparticles begin to cool. When the temperature lowers to room temperature, the gold nanoparticles are bombarded with a laser once again and they no longer heat up. The gold nanoparticles are then doped into UCNPs that have already been irradiated by a laser with a wavelength of 980 nm and a power of 500 mW. At the same time, Au-UCNPs are heated to 57 °C and immediately cooled once the irradiation is stopped. It is commonly known that at near-infrared light irradiation at a 980 nm wavelength, UCNPs can only generate green visible light without producing heat. However, after doping with gold nanoparticles, it can not only emit brighter visible light, as compared to that emitted before doping the gold nanoparticles (Figure 2) but also emit heat. The reasons for this phenomenon are as follows:

The emission bands observed at 409 nm (purple), 524 nm, 543 nm (green), and 655 nm (red) are due to the transitions of Er^3+^ ions such as ^2^H_9/2_ → ^4^I_15/2_, ^2^H_11/2_ → ^4^I_15/2_, ^4^S_3/2_ → ^4^I_15/2_, and ^4^F_9/2_ → ^4^I_15/2_, respectively (Figure 3). However, it is observed that the red and green emissions become more prominent than the purple emission which might be attributed to the cross-relaxation process from ^2^G_7/2_ to ^2^H_9/2_ levels. Furthermore, purple, green, and red lights exhibit varying degrees of enhancement. The SPR absorption peak at 520 nm coincides with the emission band of green light so the SPR vibration frequency overlaps the luminescence band of UCNPs. The coupling of the emitted light and the SPR increases the photon localized state density near the surface of Au, thereby increasing the radiation decay rate of Er^3+^ and increasing the luminous intensity. Furthermore, the SPR effect of Au produces a local electric field enhancement effect which enhances the absorption of sensitizers through electric field coupling and increases the emission intensity of the UCNPs. Moreover, the excitation wavelength of 980 nm excites the higher lying ^4^F_7/2_ level of the Er^3+^ ions and Au partially absorbs emissions coming from Er^3+^ ions that lead to the de-excitation of the fluorescence of UCNPs. This portion of the energy supplied to the surface of Au, on the other hand, radiates to the far-field with greater efficiency, increasing the fluorescence of UCNPs. The amplification of up-conversion luminescence aids in properly marking the position of a drug.

Simultaneously, gold nanoparticles enhance the luminescence intensity of UCNPs under near-infrared light with the wavelength of 980 nm and these UCNPs emit stronger light energy with the wavelength of 540 nm. This light energy further excites gold nanoparticles, resulting in the heat emission of UCNPs doped with gold nanoparticles [23].

**Figure 4:**
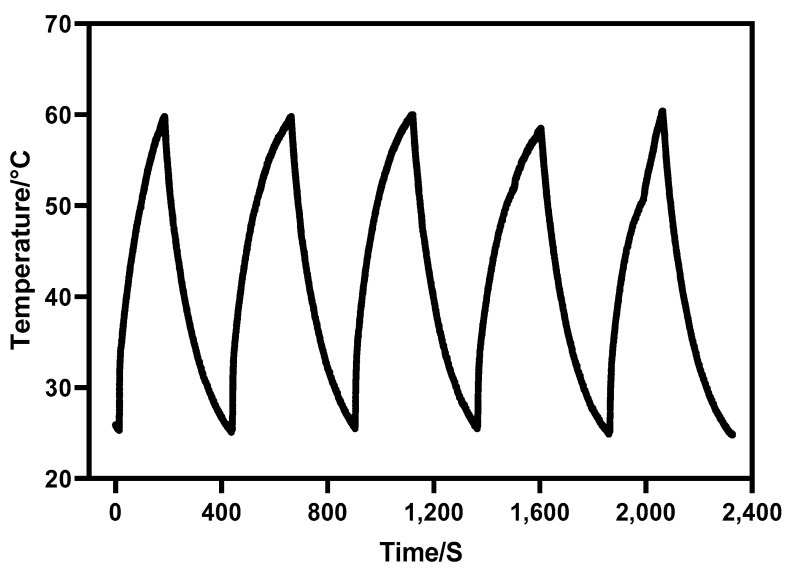
Temperature rise and drop cycle curve of 180 µg/mL of Au-UCNPs (five times).

Furthermore, under the irradiation of near-infrared light with a wavelength of 980 nm, Au-UCNPs raises the temperature to 59 °C in 250 s (Figure 4), immediately stops the irradiation, cools to room temperature in about 160 s, re irradiates for about 280 s, raises the temperature to 59 °C again, and repeats the cycle five times. Au-UCNPs still show a good photothermal effect. However, gold nanoparticles cannot be heated and cooled again under 540 nm wavelength laser irradiation (Figure 1) which shows that gold nanoparticles do not have good photothermal stability. Many experts [24] think that when gold nanoparticles are not modified in any way, the outer electrons are stimulated in the excited state, and the energy level transition happens during the process of returning to the ground state. The surface structure of gold nanoparticles alters during the energy conversion process, resulting in a mild exothermic effect when re excited.

A displacement solid solution is generated when Au nanoparticles are doped into UCNPs. Gold nanoparticles are uniformly dispersed in the hexagonal crystal structure of UCNPs, replacing some of the Er. After 980 nm near-infrared light stimulation, Au and Er contribute jointly to the hybrid orbit, enhancing the electron transition, carrier concentration, and luminescence of UCNPs. As a result, the intensity of the excitation light source of gold nanoparticles rises, thereby improving their photothermal conversion capacity. Particularly, the outer electrons of gold nanoparticles are in the excited state, and some Au nanoparticles provide hybrid orbits. Therefore, the surface structure of gold nanoparticles will hardly change which greatly enhances the photothermal stability of the gold nanoparticles.

**Figure 5:**
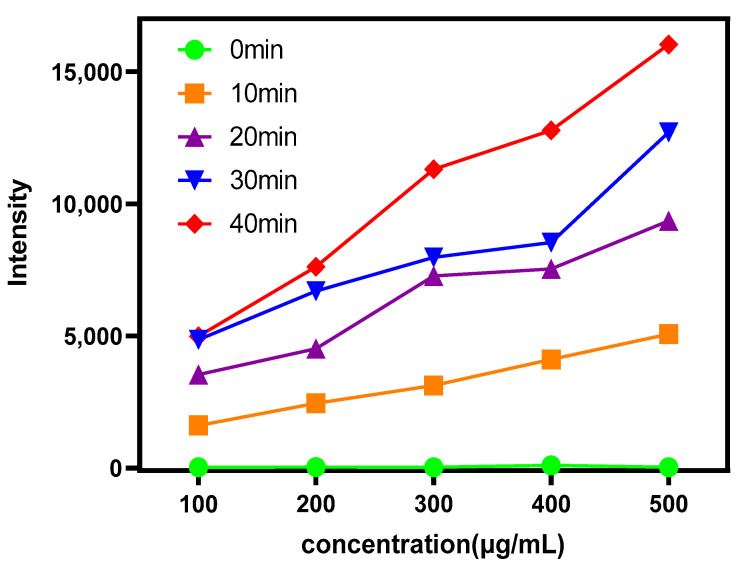
ROS generation by different concentrations of Au-UCNPs-DSPE-PEG2K was evaluated under near-infrared irradiation at 808 nm for different times.

The content of ROS released by different concentrations of Au-UCNPs-DSPE-PEG_2K_ under near-infrared light with a wavelength of 808 nm was measured by Singiet Oxygen Sensor Green (SOSG). As can be seen from Figure 5, Au-UCNPs-DSPE-PEG_2K_ hardly releases ROS without irradiation. The amount of ROS increased with the increase in Au-UCNPs-DSPE-PEG_2K_ concentration and time.

**Figure 6:**
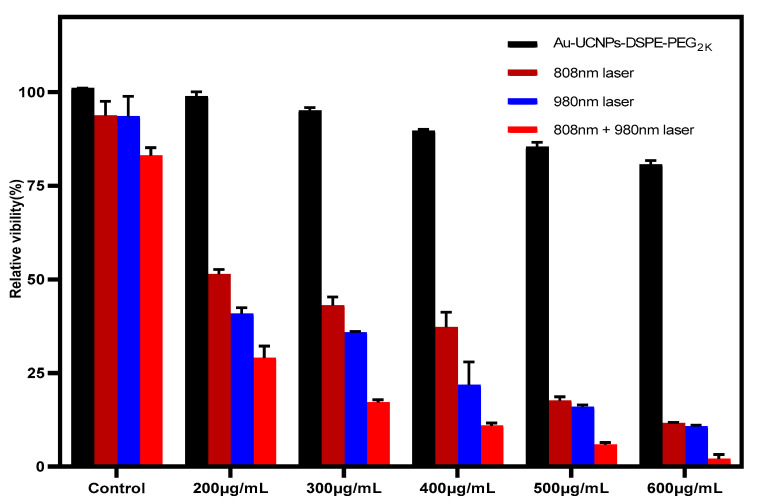
Cytotoxicity (Calculated from the absorbance measured by the microplate reader at OD = 450 nm) of different concentrations of Au-UCNPs-DSPE-PEG2K and different conditions (both the 980 nm wavelength and the 808 nm wavelength are emitted by a laser with a power of 500 mW and an irradiation radius of 12 cm).

The cytotoxicity of Au-DSPE-PEG_2K_ is tested by an enzyme labeling instrument. HeLa cells are cultivated for 4 h after dispersing modified rare-earth nanomaterials in normal saline to prepare various quantities, and their activity is assessed (Figure 6). The cell survival rate is greater than 89% if the concentration of Au-DSPE-PEG_2K_ is less than 400 µg/mL. Particularly, at 200 µg/mL, the cell survival rate is greater than 99%. According to Figure 2, a 200 µg/mL concentration of rare-earth nanoparticles not only has appropriate safety but also has a high luminous intensity. Even when the rare-earth ion concentration is as high as 500 or 600 µg/mL, cell survival remains greater than 80%. Moreover, under the irradiation of near-infrared light with the wavelength of 808 nm, ROS were produced which caused the apoptosis of cells. Under the irradiation of near-infrared light with the wavelength of 980 nm, the environment temperature increased significantly, destroying cells. When two wavelengths of near-infrared light were irradiated at the same time, the ambient temperature increased significantly and filled with ROS and the cells could hardly survive.

**Figure 7:**
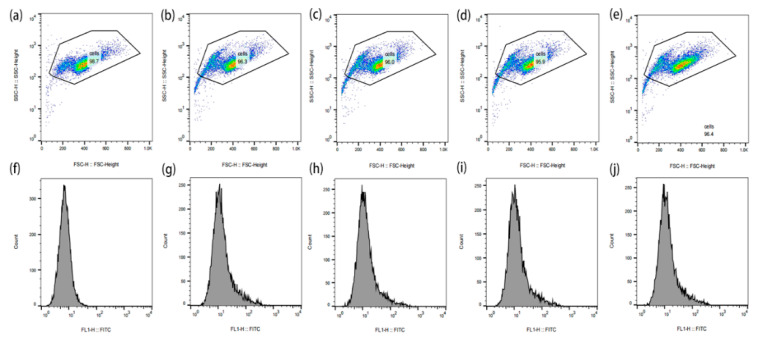
The effect of ROS produced by Au-UCNPs-DSPE-PEG2K under laser on cells and the change of fluorescence intensity after DCFH-DA staining were measured by flow cytometry (**a**): Blank; (**b**–**e**): laser for 0 min, 10 min, 20 min, and 30 min, respectively, after adding Au-UCNPs-DSPE-PEG2K; (**f**–**j**): corresponding to the fluorescence intensity of (**a**–**e**). The different color in (**a**–**e**) represents the cell concentration. FITC is that Fluorescein isothiocyanate. FSC is that forward scatter.

In order to observe the ROS produced by Au-UCNPs-DSPE-PEG_2K_ and their effect on cells more intuitively, monitoring by flow cytometry was done (Figure 7). ROS are molecules that contain hydroxyl radicals or peroxides with unpaired electrons. In healthy aerobic cells, ROS are naturally generated at a controlled rate as oxidation products of oxidative phosphorylation, oxidoreductase, or metal catalysis. However, it may be induced under some stress conditions, especially exposure to environmental oxidants and some drugs leading to oxidative stress generating ROS. Excessive ROS may destroy cellular components including DNA, proteins, and lipids, and eventually lead to cell death. Cell permeability 2, 7′—Dichlorodihydrofluorescein diacetate (DCFH-DA) is a widely used ROS indicator. The reduced non-fluorescein DCFH-DA can be oxidized by intracellular ROS and converted into fluorescent 2, 7′—Dichlorofluorescein (DCF). Therefore, Figure 7 labels intracellular ROS with DCFH-DA and detects the strength of DCF by flow cytometry.

It can be seen from Figure 7a–e that the cells initially gathered at one place. After adding Au-UCNPs-DSPE-PEG_2K_ and the laser, there were two groups in the cell community, indicating that under the laser, Au-UCNPs-DSPE-PEG_2K_ produced a large amount of ROS (Figure 5). A large amount of ROS destroyed the cells and turned the cells into fragments, resulting in two groups of cell communities, i.e., one group was cell fragments. This was also confirmed in (f–j) of Figure 7 in which can be seen, with the addition of Au and laser, the fluorescein peak shifted to the right, that is, the fluorescence was enhanced. In other words, the amount of dye DCFH-DA converted into DCF by ROS increased.

**Figure 8:**
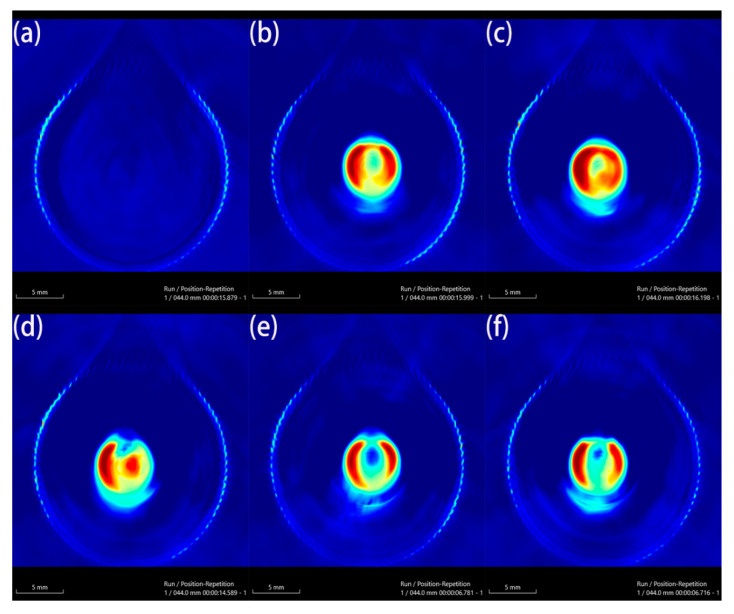
Photoacoustic imaging (PAI) of different concentrations of Au-UCNPs-DSPE-PEG2K, (**a**): 0 µg/mL, (**b**): 60 µg/mL, (**c**): 120 µg/mL, (**d**): 180 µg/mL, (**e**): 240 µg/mL, and (**f**): 300 µg/mL.

**Figure 9:**
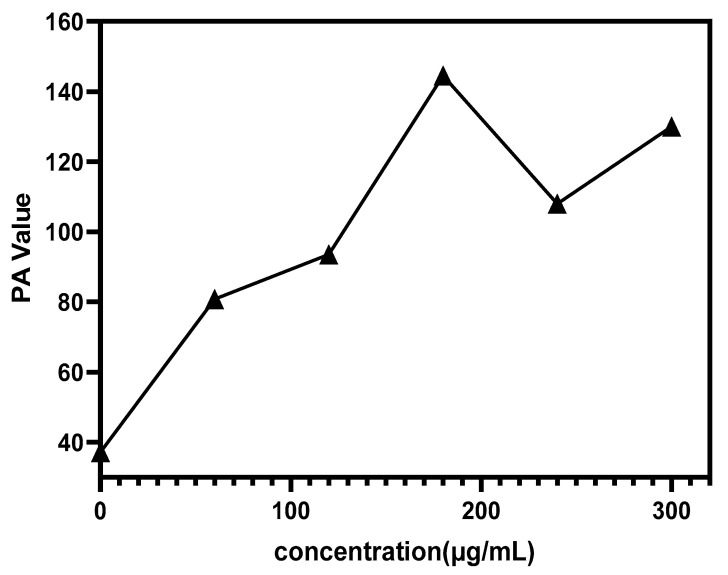
PA Value of different concentrations of Au-UCNPs-DSPE-PEG2K.

Au-UCNPs-DSPE-PEG_2K_ has excellent photoacoustic properties because of its excellent photothermal effect, characterized by photoacoustic imaging (Figure 8), and strong observed photoacoustic signals. Figure 9 shows that when the concentration of Au-UCNPs-DSPE-PEG_2K_ is 180 µg/mL, the PA(Photoacoustic) value is the strongest. Although UCNPs have X-ray attenuation characteristics and imaging ability, both CT and MRI imaging can damage biological tissues. PAI not only improves imaging ability but can also damage biological tissues with its photothermal properties. Compared with CT and MRI, the gradient is more obvious.

## 4. Conclusions

An Au-UCNPs-DSPE-PEG_2K_ multi-modality synergistic therapy device has been developed and may be utilized for photodynamic and photothermal synergistic therapy. The PA imaging experiments show that Au-UCNPs-DSPE-PEG_2K_ may be used as non-invasive imaging for both in vitro and in vivo testing, giving complete details for tumor diagnosis. Particularly, Au-UCNPs-DSPE-PEG_2K_ has better photothermal stability than gold nanoparticles and can repeat the heating and cooling process to achieve tumor treatment. These nanomaterials have exhibited low cytotoxicity, indicating their high biocompatibility for organisms. It is worth mentioning that this material can also release ROS and inhibit tumor cells to assist photothermal therapy. All these promising findings make Au-UCNPs-DSPE-PEG_2K_ nanocomposites auspicious candidates for cancer theranostics and have encouraged us to develop the integration of the diagnosis and treatment of tumors.

## Data Availability

No new data were created or analyzed in this study. Data sharing is not applicable to this article.

## References

[1] Alattar A.M., Mohammed R.A., Alwazzan M.J., Twej W.A. (2021). Dispersion of pure silica xerogel vs NaYF4- xerogel nanomaterials in silica aerogel and their effect on the optical and structural properties. Opt. Mater..

[2] Ansari A.A., Parchur A.K., Thorat N.D., Chen G. (2021). New advances in pre-clinical diagnostic imaging perspectives of functionalized upconversion nanoparticle-based nanomedicine. Coordin. Chem. Rev..

[3] Chowdhury N., Riesen N., Riesen H. (2021). Yb^3+^ and Er^3+^ Codoped BaLiF_3_ Nanocrystals for X-ray Dosimetry and Imaging by Upconversion Luminescence. ACS Appl. Nano Mater..

[4] Cordonnier A., Boyer D., Besse S., Valleix R., Mahiou R., Quintana M., Briat A., Benbakkar M., Penault-Llorca F., Maisonial-Besset A. (2021). Synthesis and in vitro preliminary evaluation of prostate-specific membrane antigen targeted upconversion nanoparticles as a first step towards radio/fluorescence-guided surgery of prostate cancer. J. Mater. Chem. B.

[5] Dong L., Zhang C., Yan L., Zhang B., Chen H., Mi X., Fu Z., Zhang Z., Zheng H. (2021). Quantifying plasmon resonance and interband transition contributions in photocatalysis of gold nanoparticle. Chin. Phys. B.

[6] Feng Z., Lin L., Wang Z., Zheng Z. (2021). Highly efficient and wide range low temperature sensing of upconversion luminescence of NaYF_4_: Er^3+^ nanoparticles: Effects of concentration of active or sensitive ions, excitation power and particle size on temperature sensing sensitivity. Opt. Commun..

[7] Zhang W., Lu Y., Zang Y., Han J., Xiong Q., Xiong J. (2021). SiO_2_ Coated Up-Conversion Nanomaterial Doped with Ag Nanoparticles for Micro-CT Imaging. Nanomaterials.

[8] Jiang J., Ren H., Huang F., Wang L., Zhang J. (2021). Refine the crystallinity of upconversion nanoparticles for NIR-enhanced photocatalysis. CrystEngComm.

[9] Jones C.M.S., Biner D., Misopoulos S., Krämer K.W., Marques-Hueso J. (2021). Optimized photoluminescence quantum yield in upconversion composites considering the scattering, inner-filter effects, thickness, self-absorption, and temperature. Sci. Rep..

[10] Zong H., Mu X., Sun M. (2019). Physical principle and advances in plasmon-enhanced upconversion luminescence. Appl. Mater. Today.

[11] Wang Y., Xu W., Lei L., Chen L., Ye R., Xu S. (2021). Photoluminescent NaGdF_4_@NaYF_4_:Ce/Tb inert-core/active-shell nanoparticles for selective and ultra-sensitive Cu^2+^ ions sensing. J. Lumin..

[12] Lu F., Zhao T., Sun X., Wang Z., Fan Q., Huang W. (2021). Rare-earth Doped Nanoparticles with Narrow NIR-II Emission for Optical Imaging with Reduced Autofluorescence. Chem. Res. Chin. Univ..

[13] Mahata M., De R., Lee K. (2021). Near-Infrared-Triggered Upconverting Nanoparticles for Biomedicine Applications. Biomedicines.

[14] Murali G., Vattikuti S.P., Kshetri Y.K., Lee H., Modigunta J.K.R., Reddy C.S., Park S., Lee S., Poornaprakash B., Lee H. (2021). Near-infrared-activated Z-scheme NaYF_4_:Yb/Tm@Ag_3_PO_4_/Ag@g-C_3_N_4_ photocatalyst for enhanced H2 evolution under simulated solar light irradiation. Chem. Eng. J..

[15] Panikar S.S., Ramírez-García G., Banu N., Vallejo-Cardona A.A., Lugo L.-F., Camacho-Villegas T.A., Salas P., De la Rosa E. (2021). Ligand-targeted Theranostic Liposomes combining Methylene Blue attached Upconversion nanoparticles for NIR activated Bioimaging and Photodynamic therapy against HER-2 positive breast cancer. J. Lumin..

[16] Bai X., Wang Y., Song Z., Feng Y., Chen Y., Zhang D., Lin F. (2020). The Basic Properties of Gold Nanoparticles and their Applications in Tumor Diagnosis and Treatment. Int. J. Mol. Sci..

[17] Tai Y., Zhang Y., Sun J., Liu F., Tian H., Liu Q., Li C. (2021). Y_2_O_3_:Yb^3+^, Tm^3+^/ZnO composite with a heterojunction structure and upconversion function for the photocatalytic degradation of organic dyes. RSC Adv..

[18] Tian Y., Liu Q., Fei E., Ye R., Chen S., Zhang J., Xu S. (2021). Structural evolution, crystallization behaviour and mid-infrared emission properties in Yb/Ho codoped oxyfluoride germanosilicate glass ceramics with varied Si/Ge ratio. Infrared Phys. Technol..

[19] Alvarez-Puebla R.A., Pazos-Perez N., Guerrini L. (2018). SERS-fluorescent encoded particles as dual-mode optical probes. Appl. Mater. Today.

[20] Xiong J., Li G., Zhang J., Li D., Pun E.Y.B., Lin H. (2021). Fluorescence regulation derived from Eu3+ in miscible-order fluoride-phosphate blocky phosphor. Nanotechnology.

[21] Zhang Y., Zhu X., Zhang J., Wu Y., Liu J., Zhang Y. (2021). Synergistic upconversion photodynamic and photothermal therapy under cold near-infrared excitation. J. Colloid Interface Sci..

[22] Zhang W., Zang Y., Lu Y., Han J., Xiong Q., Xiong J. (2022). Synthesis of Rare-Earth Nanomaterials Ag-Doped NaYF_4_:Yb^3+^/Er^3+^@NaYF_4_:Nd^3+^@NaGdF_4_ for In Vivo Imaging. Nanomaterials.

[23] Zhang Q., Iwakuma N., Sharma P., Moudgil B.M., Wu C., McNeill J., Jiang H., Grobmyer S.R. (2009). Gold nanoparticles as a contrast agent forin vivotumor imaging with photoacoustic tomography. Nanotechnology.

[24] Wei Q., Ni H., Jin X., Yuan J. (2015). Graphene oxide wrapped gold nanorods for enhanced photo-thermal stability. RSC Adv..

